# Determinants of antenatal care service utilisation in sub-Saharan Africa: an analysis of demographic and health surveys data (2015–2022)

**DOI:** 10.1186/s13690-025-01608-1

**Published:** 2025-07-17

**Authors:** Belete Achamyelew Ayele, Elizabeth Holliday, Catherine Chojenta

**Affiliations:** 1 School of Medicine and Public Health, College of Health, Medicine and Wellbeing, The University of Newcastle, Newcastle, NSW Australia; 2 Amhara Regional Health Bureau, Amhara, Ethiopia

**Keywords:** Antenatal care, Maternal health, Service utilisation, Sub-Saharan Africa

## Abstract

**Background:**

Antenatal care (ANC) is crucial for maternal and neonatal health, facilitating early complication management, health education, and promoting skilled birth assistance. Despite global ANC recommendations, implementation remains suboptimal in sub-Saharan Africa, where maternal and neonatal mortality rates remain high. Assessing ANC prevalence and its determinants can help address gaps and improve health outcomes.

**Methods:**

This study utilised data from recent Demographic and Health Surveys (DHS) conducted between 2015 and 2022 across SSA countries, using a weighted sample of 196,459 women. ANC service use during pregnancy was classified as no ANC visits, one to three ANC visits, or four or more visits. Multinomial logistic regression was used to estimate explanatory variable effects, reported as relative risk ratios with 95% confidence intervals.

**Results:**

Among participants, 11.2% received no ANC, 30.5% had one to three visits, and 58.4% attended four or more visits. ANC utilisation varied by region, with 54.7% of women in East Africa and 60.3% in West Africa receiving four or more visits. Having health insurance showed one of the strongest positive associations with ANC attendance for both one to three visits (RRR = 2.81, 95% CI: 2.37, 3.34; *p* < 0.001) and four or more visits (RRR = 2.95, 95% CI: 2.48, 3.51; *p* < 0.001). Women who did not consider obtaining permission to visit a health facility as a problem also had a higher likelihood of attending one–three visits (RRR = 1.66, 95% CI: 1.53, 1.81; *p* < 0.001) or ≥ four visits (RRR = 1.92, 95% CI: 1.77, 2.09; *p* < 0.001). Higher maternal education, longer preceding birth intervals, and an improved wealth index were significantly associated with a greater probability of attending ≥ four ANC visits (*p* < 0.001). In contrast, living in a rural area was associated with lower odds of attending ≥ four visits (RRR = 0.65, 95% CI: 0.58, 0.72; *p* < 0.001).

**Conclusion and recommendations:**

This study highlights disparities in ANC utilisation in SSA, with many women receiving insufficient or no ANC visits. Individual, household, and community-level factors, such as education, health insurance, income, geographic access, and others, strongly influence ANC service use. Strengthening maternal health insurance schemes can alleviate financial barriers, and community-based outreach programs and educational campaigns can enhance access and awareness, and improve access and continuity of care, particularly in rural or remote areas. Integrating these strategies into broader health policies and fostering collaboration between healthcare providers, policymakers, and local communities allows for narrowing existing gaps in ANC utilisation and ultimately improving maternal and neonatal outcomes across the region.

**Supplementary Information:**

The online version contains supplementary material available at 10.1186/s13690-025-01608-1.

**Table:** 

**Text box 1. Contributions to the literature**
• This research highlighted ANC service use across multiple SSA nations, leveraging recent DHS data to address region-wide service gaps.
• Unlike previous studies that dichotomised ANC, it examined three distinct categories of ANC service utilisation, offering a more nuanced perspective.
• Findings reveal that many women in SSA receive no or insufficient ANC, identified key determinants, including individual, household, and community-level factors that either hinder or facilitate optimal ANC usage.
• The findings inform that region-specific policy interventions and community-based strategies are needed to increase and sustain ANC coverage across SSA.

## Introduction

Antenatal care (ANC) is medical care provided by skilled health professionals to women during pregnancy [1]. Its core components include risk identification, prevention, pregnancy-related disease management, and health education and promotion [2, 3]. Ideally, ANC visits start in the first trimester, as most pregnancy-related complications can be predicted during first-trimester assessments [4].

Antenatal care is essential for optimising maternal and fetal health through early detection and management of pregnancy-related complications [5–9]. It also provides a platform for health education, encouraging facility-based deliveries and skilled birth attendance [10, 11]. Antenatal risk factors are also linked with adverse infant outcomes. Maternal conditions such as obesity, stress, depression, and low socioeconomic status contribute to infant adverse outcomes like preterm birth, low birth weight, and intrauterine growth restriction, which increases the risk of long-term child developmental problems and chronic diseases in adulthood [12, 13].

Despite these benefits, ANC coverage remains insufficient. According to the United Nations Children’s Fund (UNICEF), although 88% of pregnant women worldwide receive at least one ANC visit, only two-thirds complete four or more visits [14]. The World Health Organization (WHO) reported a 34% reduction in global maternal mortality between 2000 and 2020 [15]. However, sub-Saharan Africa (SSA) still faces the highest burden, with 545 maternal deaths per 100,000 live births [16], and accounts for three-quarters of the global stillbirths [17].

The WHO updated its guidelines regarding ANC visit frequency during pregnancy in 2016, increasing the recommended number of ANC contacts from four to at least eight to promote positive pregnancy outcomes and save lives [2]. However, the updated guidelines have not been widely implemented worldwide, and adherence varies significantly across regions and countries [18].

Many higher-income nations have adapted or supplemented the WHO recommendations with maternal health policies [19]. Conversely, many low-resource countries, particularly in SSA, have continued implementing the older model of at least four visits due to constrained health systems and limited resources [20, 21]. Consequently, a high proportion of pregnant women in SSA either do not attend any ANC visits or fail to complete an optimal schedule. For example, a study conducted between 2010 and 2018 revealed that approximately 13% of pregnant women in SSA do not receive any ANC visits [21], reflecting missed opportunities for preventive care. As SSA is a world region still lagging in achieving adequate ANC service use, understanding the prevalence and determinants of ANC service use in SSA could assist with implementing broad-based interventions that benefit multiple countries simultaneously while providing insight into global health initiatives. Recently published evidence was limited on this topic, except for previous studies that dichotomized outcomes within countries, not capturing variation in the number of visits when ANC visits extend beyond two categories [22–27].

To address this evidence gap, the present study used recent Demographic and Health Surveys (DHSs) data from multiple SSA countries to investigate three categories of ANC utilisation: no visits, one to three visits, and four or more visits. By examining these categories separately, the authors aimed to provide more nuanced insights into the prevalence of ANC attendance and its determinants, thereby informing evidence-based strategies for improving maternal and neonatal health outcomes in SSA.

## Methods and materials

### Study design and data source

This study employed a cross-sectional design using the most recent, nationally representative DHS from 26 SSA countries (see the list of countries and survey periods in Supplementary File 1). The DHSs are large sample, population-based surveys conducted using standardised questionnaires and methods to ensure cross-country comparability [28]. Typically, these surveys are conducted every five years. The surveys with data releases from 2015 to 2022 for each country’s most recent available release were included. This timeframe aligns with global frameworks and initiatives, including the Sustainable Development Goals (SDGs) to reduce maternal and neonatal mortality, and reflects relatively recent contexts in healthcare provision [29]. The data were accessed from the official database of the DHS program [30], after permission was granted through an online request.

### Sampling procedure

DHS surveys are hierarchical and typically follow a two-stage sampling method. In the first sampling stage, clusters, or primary sampling units (PSUs), were selected using probability sampling methods from a list of geographic areas, such as villages or census enumeration areas. In the second sampling stage, households or individuals were selected systematically or randomly from within the selected clusters. Weights were then applied to correct for unequal probabilities of selection and non-response, ensuring that each survey was representative at national and regional levels.

### Study population, sampling design, and sample size

The source population was all pregnant women and/or those who gave birth five years before each respective survey in SSA countries within the selected Enumeration Areas (EAs). Women of reproductive age (15–49 years) who had ever given birth were asked about their antenatal care, and a total weighted sample of 196,459 women was included in this study.

### Missing data handling

Missing data were assessed for all variables before analysis. DHS data are rigorously processed to minimise missing data during collection and management. In this study, missing data were addressed according to DHS guidelines. For the included variables, cases with missing information were incorporated into the denominator for all calculations, consistent with DHS methodology. Additionally, missing responses were treated as distinct to preserve the integrity of the dataset and avoid excluding observations with partial data. This approach ensured that all available and relevant data were utilised effectively, reducing potential bias while maintaining transparency in handling missing data.

### Dependent variables

Although the WHO has updated its recommendations from a minimum of four ANC visits to a minimum of eight contacts for a positive pregnancy experience [2], DHS data at the country level still categorises ANC based on a minimum of four visits [28]. Thus, the outcome variable of ANC status was classified into three categories: zero visits, one to three visits, or four or more visits.

### Independent variables

The factors that predict inadequate ANC service are multifaceted. Relevant explanatory variables for inclusion in the analysis were identified via a literature review [22–27] and incorporated based on availability within the dataset [28]. The identified, available explanatory variables were grouped into three categories: individual-level variables (maternal age, birth interval, level of maternal education, parity, husband/partner’s education status, history of pregnancy termination, opportunity to obtain permission to attend a health facility, getting money for medical treatment, distance to the health facility and respondent’s employment status); household-level variables (wealth index, media exposure, sex of household head, health insurance); and community/social variables (urban/rural residence, regional division of SSA, and country’s gross national income) level factors. Categories for each of these variables are shown in Supplementary File 2. All variables were provided in the DHS data and thus included in analyses as categorical variables.

The selection of independent variables was informed by the Socio-Ecological Model (SEM) [31], a comprehensive conceptual framework widely used to understand the multi-level influences in public health. The SEM, which assumes that the interactions between individuals and their surroundings are mutually influential, reflects the concept that individuals impact their environment and vice versa [32]. The data were extracted, cleaned, and categorised based on DHS guidelines [41] and other previous literature [33, 34], using Stata version 17 software and combined for pooled analysis and interpretation.

### Statistical analysis

Explanatory variables were summarised using frequency and percentages. Because the DHS follows a multistage sampling design, clustering at the primary sampling unit and stratification levels was accounted for using Stata’s svy commands and survey weights [28].

Analysis was performed using the survey commands (svy) in Stata, where the svyset command was applied to declare the survey design variables and characteristics. The svy prefix was then used with the mlogit command to fit multivariable multinomial logistic regression models and estimate relative risk ratios (RRR) for the multinomial outcome, considering the weighting and sampling design [28]. Multinomial logistic regression evaluates categorical membership probabilities using maximum likelihood estimation and reduces the risk of misclassification and potential biases arising from dichotomizing a naturally polytomous outcome [35]. This method allows the quantification of more nuanced relationships between predictors and multiple outcome categories, here offering more significant insights into the factors associated with different patterns of ANC use [36], which may better assist policymakers and healthcare providers in designing targeted interventions. For the multinomial outcome, the reference category was zero ANC visits, while the outcomes of interest were one to three visits and four or more visits. Model estimates were reported as RRRs with 95% confidence intervals (CI). Significance was declared at the conventional 0.05 level. Model diagnostics included collinearity checks using the variance inflation factor (VIF). The mean VIF threshold was set at five as the maximum acceptable value [37].

## Results

### Study characteristics

As recommended by DHS, sampling weights were applied to ensure representativeness, resulting in a weighted sample size of 196,459 women (Table 1). Nearly three-quarters of the women were in the 20–35 age group, and about 83% of women had longer intervals between births (≥ 36 months). A considerable number of women (41.9%) had two or fewer children, while about 19.1% had five or more children. Educational attainment among women and their partners was relatively balanced across the three educational categories, with a notable portion having attained at least primary education. Most respondents (62.7%) were employed. Approximately 60% of respondents had exposure to attending mass media, while health insurance coverage was notably low (10.3%). Most respondents resided in rural areas (64.9%). Most participants were from East and West Africa.

**Table 1:** Socio-demographic characteristics of women aged 15–49 in Sub-Saharan Africa, by number of antenatal care visits, based on demographic and health survey data collected between 2015 and 2022

Factor	Number of ANC visits					
	Zero visit	One to three visits	Four or more visits	Total		
N	21,906 (11.2%)	59,827 (30.5%)	114,726 (58.4%)	196,459 (100.0%)		
Mother’s age						
15–19	1,739 (7.9%)	5,274 (8.8%)	8,058 (7.0%)	15,070 (7.7%)		
20–35	15,091 (68.9%)	42,750 (71.5%)	84,717 (73.8%)	142,558 (72.6%)		
36+	5,077 (23.2%)	11,802 (19.7%)	21,951 (19.2%)	38,830 (19.7%)		
Preceding birth interval (months)						
< 36 Months	10,422 (56.8%)	23,725 (50.1%)	37,232 (42.9%)	71,379 (46.8%)		
≥ 36 Months	7,938 (43.2%)	23,589 (49.9%)	49,457 (57.1%)	80,984 (53.2%)		
No. children birthed						
≤ 2	7,076 (32.3%)	23,686 (39.6%)	51,510 (44.9%)	82,272 (41.8%)		
3–5	8,525 (38.9%)	23,295 (38.9%)	44,742 (39.0%)	76,561 (39.0%)		
5+	6,305 (28.8%)	12,846 (21.5%)	18,474 (16.1%)	37,625 (19.2%)		
Mother’s education level						
No education	13,994 (63.9%)	22,008 (36.8%)	30,649 (26.7%)	66,651 (33.9%)		
Primary	4,884 (22.3%)	24,329 (40.7%)	36,779 (32.1%)	65,992 (33.6%)		
Secondary+	3,027 (13.8%)	13,490 (22.5%)	47,299 (41.2%)	63,816 (32.5%)		
Husband/partner’s education level						
No education	11,957 (62.6%)	19,356 (38.6%)	27,430 (28.6%)	58,743 (35.6%)		
Primary	3,866 (20.2%)	17,987 (35.9%)	25,797 (26.9%)	47,649 (28.8%)		
Secondary+	3,271 (17.1%)	12,810 (25.5%)	42,647 (44.5%)	58,729 (35.6%)		
Ever had a terminated pregnancy						
No	19,676 (89.8%)	52,083 (87.1%)	97,139 (84.7%)	168,898 (86.0%)		
Yes	2,230 (10.2%)	7,743 (12.9%)	17,587 (15.3%)	27,560 (14.0%)		
Obtaining permission to visit the health facility						
Big problem	7,004 (32.5%)	10,239 (17.7%)	16,005 (14.6%)	33,248 (17.6%)		
Not a big problem	14,558 (67.5%)	47,737 (82.3%)	93,361 (85.4%)	155,655 (82.4%)		
Getting money for medical treatment						
Big problem	13,769 (63.9%)	32,525 (56.1%)	53,705 (49.1%)	99,999 (52.9%)		
Not a big problem	7,792 (36.1%)	25,451 (43.9%)	55,660 (50.9%)	88,904 (47.1%)		
Employment						
No	10,370 (47.3%)	22,536 (37.7%)	40,400 (35.2%)	73,306 (37.3%)		
Yes	11,536 (52.7%)	37,290 (62.3%)	74,326 (64.8%)	123,152 (62.7%)		
Wealth index						
Poor	13,906 (63.5%)	29,041 (48.5%)	41,269 (36.0%)	84,217 (42.9%)		
Middle	3,731 (17.0%)	12,544 (21.0%)	22,803 (19.9%)	39,079 (19.9%)		
Rich	4,268 (19.5%)	18,241 (30.5%)	50,654 (44.2%)	73,163 (37.2%)		
Media exposure						
No	11,798 (54.6%)	27,813 (47.4%)	38,296 (34.2%)	77,908 (40.5%)		
Yes	9,810 (45.4%)	30,837 (52.6%)	73,814 (65.8%)	114,461 (59.5%)		
Sex of household head						
Male	17,815 (81.3%)	46,869 (78.3%)	88,262 (76.9%)	152,946 (77.9%)		
Female	4,091 (18.7%)	12,957 (21.7%)	26,464 (23.1%)	43,512 (22.1%)		
Health insurance						
No	20,887 (97.3%)	51,241 (90.7%)	93,020 (87.7%)	165,148 (89.7%)		
Yes	572 (2.7%)	5,224 (9.3%)	13,075 (12.3%)	18,871 (10.3%)		
Distance to the health facility						
Big problem	11,325 (52.5%)	24,745 (42.7%)	37,590 (34.4%)	73,659 (39.0%)		
Not a big problem	10,237 (47.5%)	33,231 (57.3%)	71,775 (65.6%)	115,243 (61.0%)		
Place of residence						
Urban	4,842 (22.1%)	15,836 (26.5%)	48,335 (42.1%)	69,013 (35.1%)		
Rural	17,064 (77.9%)	43,990 (73.5%)	66,391 (57.9%)	127,446 (64.9%)		
Country region						
East Africa	6,470 (29.5%)	34,295 (57.3%)	49,263 (43.0%)	90,028 (45.9%)		
West Africa	13,212 (60.3%)	22,768 (38.1%)	54,550 (47.5%)	90,530 (46.1%)		
Central Africa	1,950 (8.9%)	2,293 (3.8%)	8,621 (7.5%)	12,865 (6.5%)		
Southern Africa	274 (1.3%)	470 (0.8%)	2,292 (2.0%)	3,036 (1.5%)		
Country’s Gross National Income						
Low income	8,352 (38.1%)	30,701 (51.3%)	51,840 (45.2%)	90,893 (46.2%)		
Lower middle income	12,950 (59.1%)	28,017 (46.8%)	57,193 (49.8%)	98,160 (50.0%)		
Upper middle income	604 (2.8%)	1,108 (1.9%)	5,694 (5.0%)	7,406 (3.8%)		

### The pooled prevalence of ANC utilisation in sub-Saharan Africa

Among the studied population, with three categorisations of ANC visit attendance status as presented in Table 2, 11.2% reported zero ANC visits, 30.5% attended one to three ANC visits, and 58.4% of women received four or more ANC visits. In East Africa, 54.7% of women attended four or more visits, compared to 60.3% in West Africa and 67.0% in Central Africa. About 75.5% of women in Southern Africa had four or more visits. The utilisation of one to three ANC visits ranged from 15.5% in Southern Africa to 38.1% in East Africa.

The data reveal a significant variation in ANC visit prevalence at the country level. Ethiopia recorded the highest prevalence of zero ANC (34.76%), while Burundi had the lowest (0.75%). Rwanda exhibited the highest prevalence of one to three ANC visits (50.33%) compared to Sierra Leone (7.77%). Finally, Ghana achieved the highest performance in four or more ANC visits (87.49%), whereas Guinea reported the lowest (34.74%) (see Supplementary File 1).

### Multinomial logistic regression analysis of ANC visits utilisation among pregnant women in sub-Saharan Africa

Multinomial logistic regression results (Table 2) show the association between explanatory variables and ANC service utilisation. The mean value of the VIF was 2.56, indicating minimal multicollinearity among the predictor variables. Compared to women aged 15–19, women aged 20–35 and over 36 were each about 50% more likely to attend four or more ANC visits than none (RRR = 1.50, 95% CI: 1.26, 1.78, and 1.25, 1.79, respectively). There was no evidence of association between age and attending one to three ANC visits. Women with a preceding birth interval of 36 months or more were more likely to attend one to three ANC visits (RRR = 1.14, 95% CI: 1.08, 1.20) and four or more ANC visits (RRR = 1.46, 95% CI: 1.39, 1.53), compared to women with a birth interval of less than 36 months. Women with three to five children had a similar likelihood of attending one to three ANC visits and four or more ANC visits compared to those with two or fewer children. However, for women with five or more children, the probability of attending four or more ANC visits decreased by an estimated 27% (RRR = 0.73, 95% CI: 0.68, 0.80).

Women with primary education were nearly twice as likely to attend one to three ANC visits (RRR = 1.94, 95% CI: 1.79, 2.09) and 2.3 times more likely to attend four or more visits (RRR = 2.30, 95% CI: 2.15, 2.52) compared to women with no education. Secondary education increased the probability of attending one to three ANC visits (RRR = 1.54, 95% CI: 1.39, 1.70) and four or more visits (RRR = 2.77, 95% CI: 2.51, 3.07). Like maternal education, partner education also influenced ANC frequency. Having a husband or partner with primary education was associated with nearly 50% higher odds of attending one to three ANC visits (RRR = 1.47, 95% CI: 1.35, 1.59) and more than 70% higher odds of attending four or more visits (RRR = 1.73, 95% CI: 1.59, 1.87). The effect of secondary or higher education was lower but still associated with one to three ANC visits and four or more ANC visits compared to those whose partners had no education. Additionally, mothers who had experienced a terminated pregnancy were more likely to fall into both the one to three and four or more ANC visits categories.

Employed women were nearly 60% more likely to attend one to three ANC visits (RRR = 1.60, 95% CI: 1.47, 1.68) and over 80% more likely to attend four or more visits (RRR = 1.82, 95% CI: 1.71, 1.94) compared to unemployed women. The household wealth index also influenced ANC attendance. Mothers from middle-income households were about 30% more likely to attend one to three and four or more ANC visits than those from poor households. Women from wealthy households had higher chances of attending one to three visits (RRR = 1.25, 95% CI: 1.13, 1.38) and four or more visits (RRR = 1.36, 95% CI: 1.23, 1.50). Women who had permission to go to health facilities and no issues with distance were more likely to attend both ANC visit frequencies than those without such access.

Contrary to other factors, mothers who did not perceive financial constraints as a significant problem were slightly less likely to attend one to three ANC visits (RRR = 0.85, 95% CI: 0.79, 0.91) and four or more ANC visits (RRR = 0.89, 95% CI: 0.83, 0.95). Women exposed to radio or TV media were likelier to attend one to three ANC visits (RRR = 1.14, 95% CI: 1.06, 1.22) and four or more visits (RRR = 1.25, 95% CI: 1.17, 1.34). Additionally, households headed by females had a slightly higher probability of mothers attending four or more ANC visits (RRR = 1.17, 95% CI: 1.07, 1.27) compared to male-headed households.

Health-insured pregnant women were nearly three times more likely to attend one to three ANC visits (RRR = 2.81, 95% CI: 2.37, 3.34) and four or more visits (RRR = 2.95, 95% CI: 2.48, 3.51) compared to uninsured women. Women who did not consider distance a major issue were 25% more likely to complete four or more ANC visits (RRR = 1.25, 95% CI: 1.17, 1.34) than those who did. Conversely, women in rural areas were less likely to attend one to three visits (RRR = 0.74, 95% CI: 0.66, 0.82) and four or more visits (RRR = 0.65, 95% CI: 0.58, 0.72).

Furthermore, regional disparities in ANC visits were highlighted, with pregnant women in West Africa being less likely to attend one to three ANC visits but more likely to attend four or more visits compared to those in East Africa. Central Africa recorded the lowest rates for one to three and four or more ANC visits. Additionally, pregnant women from lower middle-income households were less likely to attend ANC visits, with a 25% reduction in attendance for one to three visits (RRR = 0.75, 95% CI: 0.69, 0.83) and a 46% reduction for four or more visits (RRR = 0.54, 95% CI: 0.49, 0.59). No association was observed for mothers in upper-middle-income countries for one to three or four or more ANC visits.

**Table 2:** Multivariable multinomial logistic regression results on factors associated with one to three and four or more antenatal care visits (reference: no visits) among women in Sub-Saharan Africa, based on demographic and health survey data (2015–2022)

Factor	Comparison group	One to three ANC visits		Four or more ANC visits	
		RRR (95% CI)	*P*-value	RRR (95% CI)	*P*-value
Age of the mother (ref 15–19)	20–35	1.10 (0.9, 1.31)	0.301	1.50 (1.26, 1.78)	< 0.001
	36+	1.00 (0.83, 1.20)	0.992	1.50 (1.25, 1.79)	< 0.001
Preceding birth interval (ref < 36 months)	≥ 36 months	1.14 (1.08, 1.20)	< 0.001	1.46 (1.39, 1.53)	< 0.001
Parity (ref ≤ 2)	3–5	0.93 (0.87, 1.00)	0.058	0.93 (0.87, 1.00)	< 0.05
	5+	0.84 (0.77, 0.91)	< 0.001	0.73 (0.68, 0.80)	< 0.001
Maternal education (ref no education)	Primary	1.94 (1.79, 2.09)	< 0.001	2.33 (2.15, 2.52)	< 0.001
	Secondary+	1.54 (1.39, 1.70)	< 0.001	2.77 (2.51, 3.07)	< 0.001
Husband/partner’s education status (ref no education)	Primary	1.47 (1.35, 1.59)	< 0.001	1.72 (1.59, 1.87)	< 0.001
	Secondary+	1.15 (1.06, 1.26)	< 0.005	1.89 (1.75, 2.05)	< 0.001
Terminated pregnancy (ref no)	Yes	1.27 (1.17, 1.37)	< 0.001	1.38 (1.28, 1.48)	< 0.001
Getting permission to go to the health facility (ref big problem)	Not big problem	1.66 (1.53, 1.81)	< 0.001	1.92 (1.77, 2.09)	< 0.001
Getting money for medical treatment (ref big problem)	Not big problem	0.85 (0.79, 0.91)	< 0.001	0.89 (0.83, 0.95)	< 0.001
Has employment (ref no)	Yes	1.58 (1.47, 1.68)	< 0.001	1.82 (1.71, 1.94)	< 0.001
Wealth index status (ref poor)	Middle	1.31 (1.20, 1.42)	< 0.001	1.32 (1.22, 1.43)	< 0.001
	Rich	1.25 (1.13, 1.38)	< 0.001	1.36 (1.23, 1.50)	< 0.001
Media exposure (radio or TV) (ref no)	Yes	1.23 (1.15, 1.30)	< 0.001	1.37 (1.29, 1.46)	< 0.001
Sex of household head (ref male)	Female	1.12 (1.00, 1.20)	< 0.05	1.17 (1.07, 1.27)	< 0.001
Covered by health insurance (ref no)	Yes	2.81 (2.37, 3.34)	< 0.001	2.95 (2.48, 3.51)	< 0.001
Distance to the health facility (ref big problem)	Not big problem	1.14 (1.06, 1.22)	< 0.001	1.25 (1.17, 1.34)	< 0.001
Residence (ref urban)	Rural	0.74 (0.66, 0.82)	< 0.001	0.65 (0.58, 0.72)	< 0.001
Country Region (ref East Africa)	West Africa	0.59 (0.53, 0.66)	< 0.001	1.24 (1.11, 1.39)	< 0.001
	Central Africa	0.27 (0.22, 0.32)	< 0.001	0.72 (0.60, 0.87)	< 0.05
	Southern Africa	0.35 (0.18, 0.68)	< 0.005	0.45 (0.22, 0.93)	< 0.05
The country’s gross national income (ref lower-income)	Lower middle income	0.75 (0.69, 0.83)	< 0.001	0.54 (0.49, 0.59)	< 0.001
	Upper middle income	1.05 (0.65, 1.71)	0.838	1.10 (0.70, 1.72)	0.685

## Discussion

Receipt of antenatal care by pregnant women is an indicator of global women’s health monitoring and universal health coverage [29]. This study shows that at the time of relevant surveys (from 2015 to 2022), over 11% of mothers in SSA had no ANC received, and about 30% of pregnant women received partial ANC (one to three visits). These values are lower and slightly higher, respectively, than those reported in another study conducted in Papua New Guinea, where 23.6% of women received no ANC, and 25.0% received partial ANC [38]. Additionally, the proportion of women receiving four or more ANC visits in SSA was approximately 58%, which falls below the global average of 69% [14]. In East Africa, although a higher proportion of women reported attending one to three ANC visits than those in other West Africa, Central Africa, and Southern Africa subregions, a smaller proportion of women attended four or more visits, which was lower than in other subregions.

Despite its importance, ANC service utilisation in SSA remains low and is influenced by many individual household and community-level determinants. Many other studies reported different determinant factors for ANC service utilisation [22–27]. This study also identified several independent determinants for ANC service utilisation in this region. Variables including maternal age, preceding birth interval, maternal and partner’s education, history of a terminated pregnancy, sex of household head, access to health facility, employment, wealth index, media exposure, health insurance, country’s income class, residence, and country’s sub-region were significantly associated with ANC service utilisation patterns.

The study found that pregnant women aged 20–35 and those aged 36 and above were 50% more likely to attend four or more ANC visits than women aged 15–19. This finding aligns with other studies conducted in India [34], and reviews from various countries [39, 40], suggesting that women aged 20 and older may be more aware of the importance of comprehensive ANC services or have more experience navigating healthcare systems. Additionally, older women may have greater autonomy in health-related decision-making, enabling them to seek and adhere to ANC services more effectively. Strengthening health education programs targeting younger women and first-time mothers could help bridge this gap and improve early ANC utilisation.

Women with a history of terminated pregnancies were more likely to attend a range of ANC visits, a finding consistent with previous qualitative studies assessed in Ghana, Kenya, and Malawi [41]. This might be that past adverse pregnancy outcomes may prompt women to seek more antenatal care in subsequent pregnancies, which aligns with findings from a study conducted in Nepal [42]. It indicates that women who have experienced complications in previous pregnancies are more likely to attend ANC visits regularly, possibly as a precautionary measure to prevent similar outcomes.

In SSA, a positive association between longer birth intervals [36 months or more) and higher ANC visit frequency was observed, a finding supported by studies in Ghana [23] and at large scale in Africa [43, 44]. These studies found that longer birth intervals were linked to better maternal and child health outcomes, including higher ANC attendance; this might be due to better maternal health awareness and recovery between pregnancies. Additionally, extended intervals allow for more effective health education and planning. This study also found that higher parity was associated with reduced ANC utilisation. Women with five or more children showed a 27% reduction in the likelihood of attending four or more ANC visits, a finding that mirrors the results from other studies [34, 45]. This decline suggests that higher parity may be associated with decreased ANC attendance, possibly due to increased childcare.

Education equips women and their partners with the essential knowledge and awareness of the importance of ANC services. Women with primary education were approximately twofold more likely to attend ANC visits and follow-ups, and secondary education also increased this likelihood. Similarly, women whose partners have primary or secondary education significantly contributed to attending multiple ANC visits. These findings are consistent with existing literature from India [34], SSA [33], and study in multiple countries [45]. The results are plausible because educated women and their partners, being more aware of the health benefits, are more proactive in seeking healthcare. Educated mothers could adhere to ANC counselling [46] and their general health literacy [47] easily. Likewise, educated husbands might be able to challenge traditional gender roles and encourage their spouses to seek ANC visits [39].

Despite the low coverage of health insurance enrolment in SSA [48, 49], ANC follow-up is better for those with access to health insurance. In this study, pregnant women covered by health insurance had up to triple the ratio of receiving different ANC visits than non-insured, a finding consistent with a survey conducted in Papua New Guinea [38] and reviews from different countries [45, 48]. This might be because health insurance reduces out-of-pocket expenses for healthcare services [50], thereby encouraging regular ANC attendance. However, indirect costs such as transportation, time off work, and childcare can still pose barriers to attending ANC visits, even for insured women [41, 51].

Women from wealthier quintiles are more likely to attend ANC services. Women in middle and rich households’ wealth indexes enhanced ANC visit service utilisation by about half across different categories compared to women in poor households, which agrees with the previous assessment conducted in Burkina Faso [52] and Liberia [24]. Studies in China [53] and Bangladesh [54] also observed that women from the richest households were more likely to receive adequate maternal healthcare than those from poorer households. This positive association was also observed in employed women compared to unemployed women, consistent with findings from a meta-analysis conducted in Bangladesh and other low- and middle-income countries [55].

Although ease of getting the money needed for treatment is generally believed to boost ANC visits, mothers who did not perceive it as a big problem were slightly less likely to have both one to three and four or more ANC visits than those who perceived it as a big problem. The possible reasons for this are cultural beliefs and social norms, which are crucial in determining how often women seek ANC services. For instance, in some settings, such as in Nigeria, Ethiopia, and Ghana, traditional beliefs that prioritize home remedies over formal healthcare or cultural norms discouraging early pregnancy disclosure may contribute to reduced ANC utilisation. Previous research highlighted that sociocultural contexts had negative associations with ANC utilisation in some circumstances and found that women from certain ethnic groups and religious backgrounds, cultural practices, and beliefs reduce the frequency of visits despite their financial capability [56, 57]. These results explained the importance of considering cultural contexts when designing interventions to improve ANC attendance.

Media outlets, such as radio and TV, also significantly enhance the utilisation of ANC services by promoting positive health-seeking behaviors and overcoming cultural barriers [58]. In this study, women from households with access to TV or radio mass media were more likely to attend four or more ANC visits than those who did not attend media, highlighting the influence of media in shaping healthcare behaviors. Several reviews in the African [39, 59, 60] and South Asia [61] had found that regular media exposure leads to higher ANC attendance by informing women about the benefits of ANC visits and normalising them within the community. Additionally, studies in rural areas, including Malawi [61, 62],​ show that media campaigns provide accessible health information, particularly in remote locations, reinforcing public health policies and ultimately improving maternal health outcomes.

In addition, households headed by females had a positive modest relation on four or more ANC visits received. Female household heads often have greater autonomy in healthcare decision-making, a practice commonly observed in developing countries [63]. This autonomy allows them to prioritise and allocate resources to essential health services like ANC visits.

This research revealed that access to and proximity to health facilities are essential to utilising ANC services. The same findings were reported in studies conducted in Nigeria [64], and Ethiopia [25] found that women living within the nearest health facility were significantly more likely to utilize ANC services than those living farther away. Similarly, research in Indonesia [65] and reviews from various areas [66] demonstrated that proximity to health facilities was strongly associated with adherence to ANC visits.

The study found that living in rural areas is associated with lower ANC utilisation, as evidenced by studies conducted in rural communities in Kenya [67], Eritrea [68], and Nigeria [69], which consistently found a disparity in ANC visits between rural and urban areas. This difference could be due to rural women’s difficulty accessing healthcare services, including the limited availability of healthcare facilities, indicating the need for targeted interventions in rural areas.

Regarding regional variation, differences in ANC utilisation were identified, reflecting broader trends observed in SSA. Relatively, West Africa demonstrated a higher completion rate of four or more ANC visits than other regions, aligning with previous reports from the area [70]. However, women in this subregion were less likely to attend one to three visits, suggesting delayed care initiation but better adherence once the ANC was started. These differences may stem from variations in healthcare infrastructure, economic conditions, and regional healthcare policies [71]. In contrast, Central and Southern Africa consistently showed lower odds of one to three and four or more ANC visits, indicating more significant barriers to access. These disparities are linked to weak health infrastructure, long distances to facilities, and affordability challenges, particularly in Somaliland and other African low-income nations [72, 73].

Variations in healthcare infrastructure, policy implementation, and economic conditions contribute to these differences. For instance, countries with well-funded national health insurance schemes and strong primary healthcare networks tend to report higher ANC utilisation rates. In contrast, regions with fragile health systems, limited resource allocation, and conflict-related instability exhibit persistently low ANC coverage. Addressing these gaps requires region and country-specific strategies to improve healthcare access and service delivery.

Countries’ gross national income levels were also determined for pregnant women. Women were less likely to attend four or more ANC visits in lower-middle-income countries than in lower-income countries. This finding challenges the assumption that increasing income directly leads to better healthcare access and utilisation [74]. Additionally, previous assessments had suggested a positive correlation between income level and the use of ANC services [33]. The non-linear relationship between national income level and ANC utilisation may be influenced by healthcare system strength, affordability, and policy differences. Lower-middle-income countries may face reduced donor funding while lacking fully developed domestic healthcare financing, limiting access. Rising healthcare costs and out-of-pocket expenses could also create financial barriers. Additionally, variations in health policy, infrastructure, and sociocultural factors may impact ANC use, as economic growth does not always translate to improved healthcare access or utilisation. Countries like Burkina Faso, South Africa, Mali, Ethiopia, Burundi, Ghana, and others [75] exempt maternal services from payment requirements, enabling women to access ANC visits without incurring healthcare fees. Therefore, gross national income may not significantly predict maternal health once other factors are considered [76].

### Conclusion and recommendations

This study highlights the uneven utilisation of ANC services in SSA. More than 11% of pregnant women did not receive any ANC visits, and a substantial number of women did not receive even partial visits. The group receiving four or more ANC visits is suboptimal, with East Africa reporting a higher dropout rate for these visits. In this region, the pattern of ANC utilisation has not shown significant improvement since the previous assessment and lags behind the global performance [14, 21], due to various determinants.

To address these determinants, targeted interventions are needed in collaboration with local communities, healthcare providers, and policymakers. Governments across SSA countries must prioritise and implement effective maternal health policies that address systemic barriers and improve healthcare infrastructure, where access to ANC services is often limited. Global policymakers should also collaborate to mitigate maternal health problems by setting broader healthcare strategies in this region, by targeted policy measures, such as community-based health education programs, mobile clinic outreach for remote areas, or subsidized insurance schemes, ensuring universal access to quality maternal health, including ANC services, regardless of socioeconomic status. Additionally, they should strengthen the promotion of ANC services through broadcasting to comprehensively increase service utilisation, which, in turn, will encourage health facility-based deliveries.

#### Strengths and limitations of the study

This study leverages DHS data, which provides extensive, nationally representative samples and uses standardised data collection methods. This enables a broad assessment of different levels of ANC utilisation across the SSA region. However, the cross-sectional design restricts the ability to establish causality, and reliance on self-reported information can introduce recall and social desirability biases. Furthermore, because the study prioritised survey-adjusted standard errors and confidence intervals over fit statistics, the decision not to use a multilevel approach might be considered a limitation. Finally, while the WHO now recommends a minimum of eight ANC visits, the DHS data used in this study has not adopted this guideline.

## Electronic supplementary material

Below is the link to the electronic supplementary material.

**Supplementary Material 1**: **Supplementary File 1**: Countries survey period and ANC utilisation prevalence.

**Supplementary Material 2**: **Supplementary File 2**: Description of variables and their classification.

## Data Availability

No datasets were generated or analysed during the current study.

## Abbreviations

ANC: Antenatal care
CI: Confidence interval
DHSs: Demographic and health surveys
RRR: Relative risk ratios
SSA: Sub-saharan Africa
SDGs: Sustainable development goals
UNICEF: United nations children’s fund
VIF: Variance inflation factor
WHO: World health organization
