# Supplementary material for: Determinants of antenatal care service utilisation in sub-Saharan Africa: an analysis of demographic and health surveys data (2015–2022)
Source: Arch Public Health. 2025 Jul 17;83:189. doi: 10.1186/s13690-025-01608-1 (PMC12273391; doi:10.1186/s13690-025-01608-1)
Supplement: Supplementary file 1 — Supplementary Material 1: Supplementary File 1: Countries survey period and ANC utilisation prevalence. [file 13690_2025_1608_MOESM1_ESM.docx]

**Supplementary Table 1:** Survey years and prevalence of antenatal care service utilisation across 26 Sub-Saharan African countries, by number of antenatal care visits, based on Demographic and Health Survey data collected between 2015 and 2022.

| SN | Country | Survey years | ANC visits prevalence (%) | | |
| --- | --- | --- | --- | --- | --- |
|  |  |  | Zero visit | One to three visits | Four or more visits |
| 1 | Burundi | 2016/17 | 0.75 | 49.78 | 49.47 |
| 2 | Ethiopia | 2016 | 34.76 | 29.08 | 36.16 |
| 3 | Kenya | 2022 | 3.73 | 33.96 | 62.31 |
| 4 | Madagascar | 2021 | 12.03 | 29.46 | 58.51 |
| 5 | Malawi | 2015/16 | 2.16 | 47.06 | 50.78 |
| 6 | Mozambique | 2015 | 14.13 | 29.64 | 56.24 |
| 7 | Rwanda | 2019/20 | 2.09 | 50.33 | 47.58 |
| 8 | Tanzania | 2015/16 | 2.23 | 48.33 | 49.45 |
| 9 | Uganda | 2016 | 2.28 | 37.86 | 59.86 |
| 10 | Zambia | 2018 | 2.12 | 33.59 | 64.30 |
| 11 | Zimbabwe | 2015 | 5.65 | 18.08 | 76.27 |
| 12 | Benin | 2017/18 | 13.99 | 34.71 | 51.30 |
| 13 | Burkina Faso | 2021 | 1.74 | 26.21 | 72.04 |
| 14 | Cameroon | 2018 | 13.77 | 22.06 | 64.17 |
| 15 | Côte d’Ivoire | 2021 | 6.26 | 42.17 | 51.57 |
| 16 | Gambia | 2021 | 1.38 | 18.66 | 79.96 |
| 17 | Ghana | 2022 | 2.22 | 10.29 | 87.49 |
| 18 | Guinea | 2018 | 17.07 | 48.19 | 34.74 |
| 19 | Liberia | 2019/20 | 4.03 | 10.90 | 85.07 |
| 20 | Mali | 2018 | 24.40 | 33.32 | 42.27 |
| 21 | Mauritania | 2019/21 | 17.28 | 43.84 | 38.89 |
| 22 | Nigeria | 2018 | 26.12 | 17.41 | 56.47 |
| 23 | Sierra Leone | 2019 | 12.72 | 7.77 | 79.52 |
| 24 | Angola | 2015/16 | 22.51 | 20.55 | 56.94 |
| 25 | Gabon | 2021 | 8.37 | 19.47 | 72.16 |
| 26 | South Africa | 2016 | 8.10 | 13.80 | 78.10 |
