# Supplementary material for: Determinants of antenatal care service utilisation in sub-Saharan Africa: an analysis of demographic and health surveys data (2015–2022)
Source: Arch Public Health. 2025 Jul 17;83:189. doi: 10.1186/s13690-025-01608-1 (PMC12273391; doi:10.1186/s13690-025-01608-1)
Supplement: Supplementary file 2 — Supplementary Material 2: Supplementary File 2: Description of variables and their classification. [file 13690_2025_1608_MOESM2_ESM.docx]

**Supplementary Table 2:** Description and coding of variables used to examine the determinants of antenatal care service utilisation in Sub-Saharan Africa, based on Demographic and Health Survey data (2015–2022).

| Name of variable | Level (description) | Stata coding |
| --- | --- | --- |
| **Outcome variables (number of ANC visits during pregnancy)** | | |
| Multinomial outcome | Three categories of ANC visits | 0= No ANC  1= One -three ANC  2= Four and above |
| **Independent variables** | | |
| Age | Age of the mother | 0= 15-19  1= 20-35  2= 36+ |
| Preceding birth interval | Preceding birth interval in months | 0= <36 months  1= ≥36 months |
| Parity | Total children ever born | 0=≤ 2  1= 3-5  2= 5+ |
| Maternal education | Maternal level of education | 0= no education  1=Primary  2= secondary+ |
| Husband/partner's education | Husband/partner's education status | 0= no education  1=Primary  2= secondary+ |
| Pregnancy termination history | Ever had a terminated pregnancy | 1=No  2=Yes |
| Permission to go to the health facility | Obtaining permission to visit the health facility | 1= Big problem  2=Not big problem |
| Money required | Money required for medical treatment | 1= Big problem  2=Not big problem |
| Employment status | The respondent currently has employment | 0=No  1=Yes |
| Wealth index | Household wealth index status | 0=Poor  1= Middle  2= Rich |
| Media exposure | Household has radio or TV media | 0=No  1= Yes |
| Household head | Sex of household head | 1=Male  2=Female |
| Health insurance | Covered by health insurance | 0=No  Yes=1 |
| Distance | Distance to the health facility | 1= Big problem  2=Not big problem |
| Residency | Place of residence | 0=Urban  1=Rural |
| Country Region | Regional division of the countries | 0=East Africa  1=West Africa  2=Central Africa  3=Southern Africa |
| Income | Country's Gross National Income (GNI) | 1= Lower-income  2= Lower middle income  3= Upper middle income  4=Higher income |
